# Expression Profiling and Glycan Engineering of IgG Subclass 1–4 in *Nicotiana benthamiana*

**DOI:** 10.3389/fbioe.2020.00825

**Published:** 2020-07-24

**Authors:** Somanath Kallolimath, Thomas Hackl, Raphaela Gahn, Clemens Grünwald-Gruber, Wilhelm Zich, Benjamin Kogelmann, Anja Lux, Falk Nimmerjahn, Herta Steinkellner

**Affiliations:** ^1^Department of Applied Genetics and Cell Biology, University of Natural Resources and Life Sciences, Vienna, Austria; ^2^Department of Biotechnology, University of Natural Resources and Life Sciences, Vienna, Austria; ^3^Department of Chemistry, University of Natural Resources and Life Sciences, Vienna, Austria; ^4^Department Biologie, Friedrich-Alexander-Universität Erlangen-Nürnberg, Erlangen, Germany

**Keywords:** IgG subclasses, antibodies, glycan engineering, glycosylation, plants

## Abstract

IgG, the main serum immunoglobulin isotype, exists in four subclasses which selectively appear with distinctive glycosylation profiles. However, very little is known about the biological consequences mainly due to the difficulties in the generation of distinct IgG subtypes with targeted glycosylation. Here, we show a comprehensive expression and glycan modulation profiling of IgG variants *in planta* that are identical in their antigen binding domain but differ in their subclass appearance. While IgG1, 2, and 4 exhibit similar expression levels and purification yields, IgG3 is generated only at low levels due to the *in planta* degradation of the heavy chain. All IgG subtypes are produced with four distinct N-glycosylation profiles, differing in sugar residues previously shown to impact IgG activities, i.e., galactosylation, sialylation and core fucosylation. Affinity purified IgG variants are shown to be fully assembled to heterodimers but display different biochemical/physical features. All subtypes are equally well amenable to targeted glycosylation, except sialylated IgG4 which frequently accumulates substantial fractions of unusual oligo-mannosidic structures. IgG variants show significant differences in aggregate formation and endotoxin contamination which are eliminated by additional polishing steps (size exclusion chromatography, endotoxin removal treatments). Collectively we demonstrate the generation of 16 IgG variants at high purity and large glycan homogeneity which constitute an excellent toolbox to further study the biological impact of the two main Fc features, subclass and glycosylation.

## Introduction

IgG is the main immunoglobulin class that is induced during an immune response against foreign antigens. Comparable to the other antibody classes, IgGs are characterized by a bifunctional action mode. While Fab region precisely recognize an antigen, the Fc domain conveys a wide range of effector functions that modulate various aspects of innate and adaptive immunity. Fc mediated activities are mainly initiated upon interactions with the various types of Fc gamma receptors (FcγRs), a process that is largely determined by the structural heterogeneity of the IgG Fc domain. The two main features that generate a spectrum of Fc phenotypes are modulation of Fc-glycosylation along with differences in the amino acid sequence among the IgG subclasses. The 30–35 N-glycan structures usually detected on serum Fc-IgG are of biantennary complex type, most of them being core-fucosylated. Three forms are dominant, glycans terminating either with GlcNAc (GnGnF, also known as G0F), a single or two β1,4 linked galactose residues (AGnF and AAF; also known as G1F and G2F). Notwithstanding all IgG subclasses have their typical glycan signature which are used for activity modulation (Alter et al., 2018). Recent studies have clearly documented the selective appearance of IgG subclasses with distinctive glycosylation profiles not only during different ontogenetic stages, but also during infections and inflammatory diseases (Alter et al., 2018). However, the biological consequences are poorly understood. Especially three sugar residues, i.e., galactose, sialic acid and core fucose, have been shown to modulate the pro-inflammatory and anti-inflammatory effects of IgGs (Seeling et al., 2017). Most of these studies refer to IgG1 and very little is known about the impact of glycosylation on the activity of the other three IgG subclasses.

Major obstacles in studying the effect of Fc subclass glycosylation in more detail are the difficulties in separating of individual subclasses from the serum and producing distinct glycoforms. Several cellular engineering approaches are underway and novel genome editing techniques allow for efficient targeted genome-wide engineering of the glycosylation pathways (Yang et al., 2015; Narimatsu et al., 2019; Dekkers et al., 2016). Plants are at the forefront in this context. As higher eukaryotes they are able to synthesize complex biantennary N-glycans typical for IgG antibodies. However, plants lack the large glycome diversifications observed in mammalian cells, which complicates a controlled manipulation. Shortcomings of plants are the presence of plant specific core xylose and fucose and the plant‘s inability to elongate GlcNAc terminating structures (e.g., by β1,4 galactose and sialic acid). The plant specific sugar residues have been successfully eliminated by various knock out/down methods allowing for the production of antibodies with human type GnGn structures (Strasser et al., 2008; Jansing et al., 2019). Moreover, introducing foreign glycosyltransferases (β1,4 galactosyltransferase and α2,6 sialyltransferase) along with the sialic acid biosynthetic pathway resulted in the production of antibodies with galactosylated and sialylated N-glycans, respectively (Montero-Morales and Steinkellner, 2018). It appears that plants exhibit an unusual high flexibility in modulating their endogenous glycosylation machinery toward human type structures. This makes them suitable hosts for approaching bottlenecks in antibody production and glycosylation.

Here we aimed to introduce a plant-based expression platform that meets the current shortcomings in IgG subclass research. We used Rituximab as a model to monitor in a side-by-side study the expression, purification and glycan engineering of IgG subclass 1–4 in *Nicotiana benthamiana*, a plant species widely used for recombinant protein expression. We generated Rituximab IgG variants with identical antigen binding features but differing in their subclass appearance (RxIgG1-RxIgG4) and transiently expressed them in *Nicotiana benthamiana*. All four subclasses were subjected to glycan engineering approaches to generate four distinct glycosylation profiles frequently observed to modulate IgG activities. We show that IgG subclasses are generated differentially well in plants and exhibit distinct biochemical/biophysical features albeit all subclasses are equally well amenable to glycan engineering approaches.

## Materials and Methods

### Cloning of Rituximab IgG Subclasses

Rituximab heavy and light chain (HC 1356 bp; LC 645 bp) codon optimized for *Nicotiana benthamiana* were subcloned into the MagnICON^®^ vectors TMVα and PVXα to generate TMVαRxIgG1HC (*RxIgGHC1*) and PVXαRxIgGLC (*RxLC*). IgG subclasses (IgG2-4) were generated in two steps: (i) a 363-bp HC variable region was amplified from *RxIgG-HC1* using primer pair (RxFvHC F2/RxFvSOE R2) and HC constant regions were amplified from different subclasses (Lux et al., 2013) with primer pair (RxFvSOE F/7B4IgGHC R1); (ii) full-length *HC* fragments that carry the identical variable domain but different subclass sequences were generated using splicing by overlap extension polymerase chain reaction (SOE-PCR) with primer pair (RxFvHC F2/7B4IgGHC R1). The primers also introduce *Bsm*BI restriction site and flanks the fragment with *Bsa*I restriction site to facilitate cloning into MagnICON^®^ vector (Marillonnet et al., 2005). The full-length *HC* fragments of *RxIgG-HC2* (1344-bp); *RxIgG-HC3* (1497-bp) and *RxIgG-HC4* (1347bp) were cloned into MagnICON^®^ TMV-based vector containing barley alfa amylase signal peptide resulting in TMVαRxIgGHC2-4 (*RxIgGHC2-4*) and transferred to Agrobacteria (strain GV3101 pMP90). For sequence information see Supplementary Figure 1A and Table 1.

**TABLE 1:** Yields of RxIgG after Protein A/G purification (before SEC).

**RxIgG subtype**	**Yield (μg/g leaf)**
IgG1	90–170
IgG2	60–80
IgG3	6–16
IgG4	50–160

### Agroinfiltration, Protein Extraction, SDS-PAGE and Immunoblotting

The *Nicotiana benthamiana* glyco-engineered plants ΔXTFT and ΔXTFT^GalT^ (Strasser et al., 2008; Schneider et al., 2015) were grown in a plant chamber at 24°C, 60% humidity with a 16 h light/8 h dark photoperiod. Leaves of 4,5 weeks old plants were used for infiltration. Agrobacteria transformed with *RxIgGHC1-4* and *RxIgGLC* were grown in liquid culture at 29°C for 24 h, centrifuged (5 min at 3000 g) and resuspended in infiltration buffer (10 mM MES pH 5.6; 10 mM MgSO4) to a final optical density (OD_600_) of 0.1 and mixed in a 1:1 ratio for infiltration as described (Castilho et al., 2010). Only syringe-based infiltrations were done. Usually fully expanded 2–3 (middle) leaves were infiltrated, depending on the susceptibility of the agrobacterial suspension cultures.

For the generation of GnGnF structures an agrobacteria strain (strain UIA-143) carrying a binary vector that expresses Zea mays core α1,3-fucosyltransferase (FUT11) along with RxIgGHC1-4 and RxIgGLC (all OD_600_ of 0.1) were mixed in a 1:1 ratio and infiltrated into ΔXTFT plants. To modulate plant glycosylation toward the synthesis of terminally sialylated N-glycans, seven agrobacteria strains (strain UIA-143) carrying binary vectors necessary for the *in vivo* synthesis of sialylated N-glycans (GNE, NANS, CMAS, CST, ST-GalT, ST, FUT11) were co-expressed along with RxIgG1-4 and RxIgGLC. All suspension cultures were diluted to OD_600_ 0.1 and mixed in a 1:1 ratio prior infiltration (Castilho et al., 2010).

Four days post infiltration (dpi), leaves were collected and flash-frozen in liquid nitrogen. Total soluble proteins (TSPs) were extracted from infiltrated leaves in extraction buffer (0.5 M NaCl, 45 mM Tris, 1 mM EDTA, and 40 mM ascorbic acid; pH 7.4) in a ratio of 1:2 w/v (fresh leaf weight/buffer).

SDS-PAGE analyses were performed in 12% gel under reducing or non-reducing conditions. Gels were stained with Coomassie Brilliant Blue R 250 staining (Carl Roth GmbH + Co., KG) or used for Immunoblotting using anti-human IgG (1:5,000 Promega anti-hIgG-HRPO, W4031).

### Purification of RxIgG Subclasses

The recombinant RxIgG1, 2, and 4 were purified by affinity chromatography using protein A, and for RxIgG3 protein G was used (Protein A/G SepharoseTM Fast Flow, GE Healthcare, suitable for bioprocess medium). Antibodies were eluted with 0.1 M Glycine/HCl (pH 2.5) and neutralized with 1 M Tris (pH 9). Purified antibodies were dialyzed overnight against PBS and yield determined by spectrophotometer (NanoDrop^TM^ 2000, Thermo Scientific). RxIgG3 and RxIgG4 variants were subjected to preparative size exclusion chromatography (SEC) using a HiLoad 16/600 Superdex 200 prep grade column (GE Healthcare). The column was equilibrated with 1.5 CV running buffer (1xPBS, 200 mM NaCl, pH 7.4) before loading the sample. All steps took place at a flow rate of 0.8 mL/min (Montero-Morales et al., 2019). The fractions corresponding to the monomeric peak collected and concentrated with Amicon centrifugal filters, MWCO 10,000 kDa (Merck Millipore).

### LPS Removal

LPS contamination of purified IgGs was determined using LAL endotoxin assay kit (Toxin Sensor^TM^ Endotoxin detection system, Genscript) according to the manufacturer’s protocol before SEC. LPS removal was carried out with a two-phase extraction method. 10% Triton X-114 was mixed with IgG samples in the ratio of 1:10 (v/v), vortexed for 30 s, incubated on ice for 15 min followed by incubation at 42°C for 10 min. The milky white solution was then centrifuged using a swing out rotor for 5 min, 2000 g at room temperature. The top phase containing LPS free protein sample was collected, the bottom phase containing Triton X-114 with LPS was discarded. The procedure was repeated 3–5 times.

### Glycan Analysis

For glycopeptide analysis, we followed the LC-ESI-MS (LC-ESI-MS/MS)-based protocol as described previously (Loos et al., 2014). Briefly, the heavy chain was excised from an SDS-PAGE, S-alkylated with iodoacetamide, proteolytically digested using trypsin (Promega), and the peptide mixture was subsequently analyzed using a Dionex Ultimate 3000 system directly linked to a Q-TOF instrument (maXis 4G ETD, Bruker) equipped with the standard ESI source (end plate offset 500V; capillary 4,500V; dry gas (nitrogen) 5.0 L/min; dry temp 200°C) in the positive ion, data dependent acquisition mode. MS-scans were recorded (range: 150–2,200 m/z, spectra rate: 0.5 Hz). Instrument calibration was performed using an ESI calibration mixture (Agilent). For peptide separation, a Thermo BioBasic C18 separation column (5 μm particle size, 150 × 0.32 mm) was used. MS/MS spectra were used for the verification of the glycopeptides by detection of oxonium ions HexNAc (*m*/*z* = 204.1), Hex + HexNAc (*m*/*z* = 366.1) and the unique Y1 ion (peptide + HexNAc). Glycopeptides were identified as sets of peaks consisting of the peptide moiety and the attached N-glycan varying in the number of HexNAc units, hexose, deoxyhexose, sialic acid and pentose residues. The theoretical masses of these glycopeptides were determined with a spread sheet using the monoisotopic masses for amino acids and monosaccharides. N-glycosylation site occupancy was calculated from the ratio of deamidated to unmodified peptide upon N-glycan release with 0.15 mU of PNGase A (Europa Bioproducts) overnight at 37°C.

## Results

### Monitoring of RxIgG Subtype Expression

**FIGURE 1:**
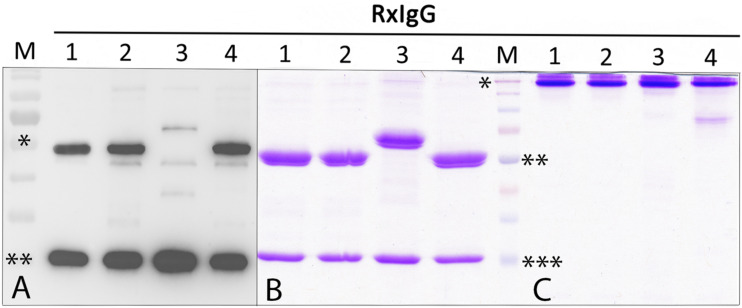
Expression and purification profiling of RxIgG1-4. **(A)** Western blot analysis of total soluble proteins (TSP) extracted from ΔXTFT plants infiltrated with RxIgG1–4 (in each lane approx 50 μg TSP was loaded); M: marker: ^∗^ = 55, ^∗∗^ = 25 kDa; **(B)** SDS-PAGE of purified RxIgG1-4 under reducing, and **(C)** non-reducing condition (4 μg IgG was loaded in each lane). Marker (M) ^∗^ = 160 kDa, ^∗∗^ = 55 kDa, ^∗∗∗^ = 25 kDa. IgG 3 and 4 underwent an additional SEC purification step.

For recombinant IgG subtype expression, we used the monoclonal antibody Rituximab (RxMab), a clinically approved human IgG1 variant and the standard care in a variety of CD20-overexpressing cancer types (Pierpont et al., 2018). Collectively, four plant-based expression vectors were generated that carried the identical variable region of RxMab HC, but differed in their HC constant domains, representing subtypes RxIgG1-4 (*RxIgGHC1-4*, Supplementary Figure 1A). For the expression of LC the sequence of RxMab LC was used (*RxIgGLC*). All four IgG variants were transiently expressed in *Nicotiana benthamiana* glycosylation mutant ΔXTFT, synthesizing human type bi-antennary complex N-glycans lacking plant-specific xylose and core fucose residues (Strasser et al., 2008). Plant leaves were harvested 4 days post infiltration (dpi) and expression levels monitored. Immunoblotting using anti IgG antibodies revealed the presence of two strong signals corresponding to an apparent molecular mass (MM) of 55 (65 kD for IgG3) and 25 kDa, as expected for HC and LC (Figure 1A). While expression was approximately the same for RxIgGHC1, 2, and 4, detection of IgG-HC3 was significantly lowered (>10 times), with additional signals at MM of 40 kDa which most probably refer to HC degradation. In all samples an unspecific faint signal corresponding to MM of 50 kDa was visible.

### Glycan Engineering and Purification

Within this project we aimed to generate IgG subtypes with distinct Fc-glycosylation profiles. We focused on the production of glycovariants that differ in glycan residues previously shown to impact the biological activities of IgGs, i.e., core fucose, β1,4 galactose and α2,6 sialic acid (Supplementary Figure 1B). Thus, the following structures were envisaged GnGn (terminating with GlcNAc residues, lacking fucose/xylose), GnGnF (terminating with GlcNAc residues, carrying core fucose), AA: elongated β1,4 galactose, and NaNaF (elongated with α2,6 sialic acid and core fucose). For the production of GnGn structures appropriate IgG subtype vectors were expressed in the *N. benthamiana* glycosylation mutant ΔXTFT. For the generation of GnGnF structures a binary construct carrying α1,3 fucosyltransferase (FUT11) was included in the infiltration mix (Castilho et al., 2015). Transgenic ΔXTFT plants that overexpress modified β1,4 galactosyltransferase (ΔXTFT^GALT^) (Schneider et al., 2015) were used to generate IgGs with AA structures. Lastly, for the synthesis of NaNaF structures seven foreign genes working along the sialic acid pathway were added to the infiltration mix (Castilho et al., 2010, 2013). Antibodies were harvested 4 dpi for subsequent purification.

Glycovariants of RxIgG1,2, and 4 were purified by Protein A affinity chromatography, for RxIgG3 Protein G was used. While the major fraction of RxIgG1 and 2 eluted with a single peak, RxIgG3 and 4 exhibited additional peaks. Thus, RxIgG3 and 4 were subjected to size exclusion chromatography (SEC) to isolate assembled monomeric variants (Supplementary Figure 2). It should be noted that the IgG recovery rate upon SEC treatment was only about 50%. The purified RxIgG variants were monitored on SDS-PAGE analysis and a typical result of IgGs produced in ΔXTFT is shown in Figures 1B,C. Under reducing conditions all four subclasses exhibited two major bands corresponding to the MM of the IgG heavy and light chains (55 and 25 kDa, respectively, Figure 1B), irrespective of their glycosylation profile. As expected RxIgG3HC appeared as 65 kDa band. Notably, in various RxIgG3 samples an extra band corresponding to MM of 40 kDa appeared. This signal which refers most probably to degraded HC was not detected after SEC (Supplementary Figure 2B). In all samples HC appeared as a double band, the smaller fraction most likely representing the non-glycosylated version, a frequently observed feature of plant produced IgGs (Castilho et al., 2018). Non-reducing SDS-PAGE showed a predominant signal at MM > 160 kDa (Figure 1C) indicating full assembly of IgG variants without the presence of any obvious degradation products or impurities. An exception is RxIgG4 which exhibited a weak signal at position 80 kDa, which could not be eliminated upon SEC treatment.

Each subclass and glycoform was generated 3–5 times and the obtained yields are listed in Table 1 (for better comparison yields before SEC are shown). While RxIgG1, 2 and 4 gave consistent yields between 50–170 μg/g leaf material, the yields of RxIgG3 were significantly lower, with maximum purification levels of 16 μg/g leaf material. This is in accordance with the reduced HC levels detected in Western-blotting. Highest IgG levels were obtained with RxIgG1 and 4. Note, variation in purification yields within subtypes are not connected with the glycosylation status, obvious reasons are plant handling susceptibility of leaves for syringe based agroinfiltration and some variation in plant growth.

### Glycan Analysis

**FIGURE 2:**
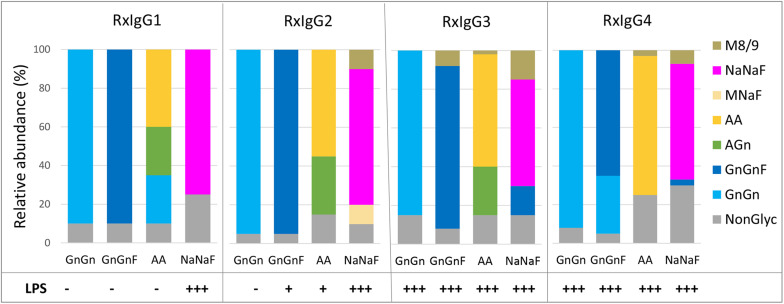
MS based analyses of the glycosylation status of plant produced RxIgG 1–4. All four subclass were expressed in ΔXTFT (GnGn version), coexpressed with FUT11 in ΔXTFT (GnGnF version), ΔXTFT^GalT^ (AA version) and ΔXTFT co-expressing seven genes for *in planta* protein sialylation (NaNaF). Glycovariants were named after their main glycoform. Non-glyc: non-glycosylated HC. Bars represent the relative abundance (%) of glycoforms present in each IgG variant (for detailed information, see also Supplementary Table 2). Bottom: detection of lipopolysccharides (LPS) in each glycovariant: – <0,1 EU/ml, +0,1–0,25 EU/ml, +++ >0,5–EU/ml.

To evaluate the glycosylation status of IgG variants liquid chromatography-electrospray ionization-tandem mass spectrometry (LC-ESI-MS/MS) was performed. First, Fc glycosite occupancy rates were monitored and the profiles exhibited that all variants were under-glycosylated (20–40%) (Figure 2). Unfortunately, control of this unwanted feature was not possible. We did not observe a connection of under-glycosylation with subclass or glycosylation engineering.

The glycosylation profiles of all four RxIgG subclasses are summarized in Figure 2 (and Supplementary Table 2). RxIgGs produced in ΔXTFT plants exhibited virtually exclusively GnGn structures. Co-expression of FUT11 resulted in the generation of mainly GnGnF structures, with minor amounts of GnGn. RxIgG4 provides an exception with 30% unprocessed GnGn structures. However, it should be noted that in small scale experiments where only one or two leaves were infiltrated frequently (but not always) >90% GnGnF was achieved, demonstrating that by optimal conditions a complete processing is feasible. However, we were not able to exactly determine such “optimal” conditions. IgG expressed in ΔXTFT^GalT^ plants give rise to varying amounts of galactosylated structures (65-85%) and our attempt toward IgG subtype sialylation resulted in the synthesis of partially sialylated structures (60-75%). Please note that due to the special structural feature of the IgG-Fc domain, the synthesis of sialylated structures lacking core fucose is not possible (Castilho et al., 2015). Interestingly, single plants from time to time show exclusively AA or NaNaF structures, demonstrating that a complete processing toward targeted structures is feasible. However, we were not able to tightly control the parameters for homogeneous galactosylation and sialylation when we upscaled the procedure and combined infiltrated leaves of >10 plants. Also, galactosylated and sialylated RxIgG4 frequently carried substantial amounts of mannosidic structures (30–60%). These unwanted glycoforms could be significantly reduced by SEC (Supplementary Figure 3).

### LPS Content of IgG Subtypes

A persistent problem associated with the production of recombinant proteins when used in animal studies or therapeutic applications is the presence of endotoxins in purified protein preparations. In this respect, the presence of lipopolysaccharides (LPSs) is a primary concern for the manufacturers, particularly when bacterial expression systems are used (Mack et al., 2014). Here we evaluated the presence of LPS on all IgG variants and found differential contaminations (Figure 2). While on IgG1 and 2 no or only minor traces of LPS were detected on GnGn, GnGnF and AA glycovariants (<0,1–0,25 EU/ml) there were significant amounts on the sialylated versions (>0,5−EU/ml). Surprisingly, all IgG3 and four subtypes, irrespective of their glycosylation status, were heavily contaminated with LPS (>0,5−EU/ml). Notably, LPS could be efficiently removed (<0,1 EU/ml) upon repeated two-phase extraction using Triton X-114 without diminishing antibody yield.

## Discussion

Here we evaluated a *Nicotiana benthamiana* based expression platform for the generation of RxIgG subclasses with targeted glycosylation profiles. This is highly relevant as recent studies demonstrate that individual IgG subclasses show a differential requirement for Fc-glycosylation and question the concept that some IgG subclasses (e.g., IgG2 or IgG4) are functionally inert with respect to triggering Fc-dependent effector functions (Lux et al., 2014; Kao et al., 2015). Also, IgG2, 3, and 4 antibodies are being developed for therapeutic applications to replace IgG1, without an in-depth understanding of how glycosylation impacts their activity (Rosner et al., 2013; Lux and Nimmerjahn, 2015; Zou et al., 2016).

We show a comprehensive expression and glycan engineering profile of RxIgG variants that are identical in their antigen binding domain but differ in their subclass appearance. While RxIgG1, 2, and 4 exhibited similar expression levels and purification yields, full-length RxIgG3HC was detected at very low levels in immunoblotting which correspondingly resulted in low levels of purified assembled protein (>10 times less). IgG3 is together with IgG4 the less abundant subclass in human serum (4–8%) (Vidarsson et al., 2014). The reasons for heavy IgG-HC3 degradation *in planta* are currently unknown, however, this subclass stands out from the other IgG subclasses because of its elongated hinge region. Interestingly, the repeat regions within the IgGHC3 hinge were found to partially carry core-1 type O-glycans (Plomp et al., 2015), structures that are not synthesized in plants. The function of the O-glycans found on the IgG3 hinge region has not been investigated in detail, but previous findings suggest that hinge glycosylation might prohibit proteolytic degradation (Plomp et al., 2015). Therefore, a lack of IgG3 O-glycosylation *in planta* could result in the observed heavy chain degradation. Engineering of human type core 1 type O-glycans has been reported previously by our group (Dicker et al., 2016) and might be a strategy to the increase expression/stability of this IgG subclass *in planta*.

Notably, all subtypes were partially under-glycosylated, an observation reported earlier at plant produced glyco-proteins (Castilho et al., 2018). A possible strategy to overcome this unwanted feature might be the co-expression of bacterial oligosaccharyltransferase which increases protein N-glycosite recognition in plants (Castilho et al., 2018). With regards to N-glycan engineering all four RxIgG subclasses are similarly accessible to the synthesis of defined glycan structures, yet not all forms are generated in equal efficiencies. While the generation of GnGn and GnGnF structure was straight forward, the synthesis of galactosylated and sialylated carbohydrates was more challenging. Although we were able to generate these glycoforms up to 75%, they were lagging behind core fucosylation, where constant levels of >90% were achieved. Also, regarding the synthesis of AA and NaNaF structures we observed batch to batch variations of up to 30%. An impediment in the *in planta* generation of β1,4 galactosylated proteins might be the presence of highly active β1,4 galactosidases (Kriechbaum et al., 2019). Although IgG-Fcs are less prone to these enzymes when compared to other proteins, they are partial vulnerable. Of note, capping β1,4 galactose by sialic residues prevents β1,4 galactosidase action (Kriechbaum et al., 2019). Variation in the synthesis of sialylated structures by the transient expression approach applied in this study was not surprising since it needs the coordinated action of nine foreign genes *in planta*, which is *per se* afflicted with inconsistencies. The use of transgenic plants that express the sialylation pathway may be a solution and has been reported previously (Kallolimath et al., 2016). However, such plants are currently only available in limited numbers since they exhibit a significant reduction in seed production compared to WT plants for so far unclear reasons (Kallolimath et al., 2016). For studies where sialylated structures >80% are required to reveal biological consequences post purification approaches like incubation with *Sambucus nigra* agglutinin, a lectin that binds preferentially to β1,4 galactose linked sialic acid, may be applied (Kaneko et al., 2006).

It also should be noted that despite keeping syringe infiltration procedure as constant as possible it is prone to inconsistencies due to handling by different operators. Also, plants were propagated and maintained manually (watering, fertilization, etc.) resulting in phenotype variation which might impact susceptibility of plants to syringe infiltration. Both effects are mainly associated to lab-scale applications but could be better controlled using a fully automated plant growth and infiltration systems (e.g., vacuum infiltration) for large-scale production. This may contribute to diminish variability on expression profiles. Harvesting single leaves (as performed by small scale purification) shows in some cases full sialylation or galactosylation. Currently we do not know under which conditions such glycans are synthesized, however, intrinsic features of plants may also impact as the same infiltration mix is delivered to different plants. A phenomenon that is frequently observed is reduced susceptibility of bacterial liquid to plant leaves, despite side by side propagation of plants. Leaves that could be infiltrated by a single infiltration event usually show more homogeneous glycosylation than leaves that need several treatments to disperse the liquid throughout the leave. The latter may induce severe plant defense/repair mechanisms which may impact the secretory pathway and impair glycosylation.

An important quality control in the context of *in vivo* studies of recombinant proteins is the examination of detrimental substances like LPS. While this is a major concern in bacterial expression systems, not much information on this topic is known for plant produced recombinant proteins. We observe differential LPS contamination of RxIgG subtypes, with no clear pattern. At this moment we do not know where LPS contamination occurs, *in planta* or along the purification chain. Importantly, Triton X-114 treatment is a powerful approach to efficiently remove the toxic substance without loss of protein yield. Interestingly, all sialylated mAbs were heavily contaminated with LPS, which could be a consequence of the many bacterial strains used in this infiltration mix.

Several mAbs of different subclasses are currently used as therapeutic molecules such as the IgG2 (Panitumumab) and IgG4 (Pembrolizumab and Nivolumab) and many more are under investigation (Kaplon et al., 2020). Moreover, systematic serological profiling (e.g., after vaccination) exhibit a discerning appearance of IgG subtypes carrying individual glycosylation signatures (Ackerman et al., 2017). These data point to a highly selective tuning of the “antibody-ome” to achieve optimized activities. Our data on IgG subtype glycosylation allow to perform fundamental studies toward a more profound understanding of coordinated aspects of humoral immune responses. This helps to select Ab formats exhibiting optimized activities for therapeutic applications.

Collectively, we were able to show the tremendous power of the *Nicotiana benthamiana* based expression approach for the generation of RxIgG subtypes with controlled glycosylation profiles. We exhibit shortcomings and provide solutions. We now have a tool in hand that allows the systematic analysis of the functional consequences determined by IgG subclass and glycosylation *in vitro* and *in vivo*.

## Data Availability Statement

The raw data supporting the conclusions of this article will be made available by the authors, without undue reservation, to any qualified researcher.

## Author Contributions

HS, FN, AL, and SK designed the research. SK, TH, RG, WZ, AL, and BK performed the research. HS, AL, SK, TH, RG, WZ, CG-G, and BK analyzed the data. HS, AL, FN, and SK wrote the manuscript. All authors contributed to manuscript revision, read, and approved the submitted version.

## Conflict of Interest

The authors declare that the research was conducted in the absence of any commercial or financial relationships that could be construed as a potential conflict of interest.
