# Supplementary material for: Expression Profiling and Glycan Engineering of IgG Subclass 1–4 in Nicotiana benthamiana
Source: Front Bioeng Biotechnol. 2020 Jul 24;8:825. doi: 10.3389/fbioe.2020.00825 (PMC7393800; doi:10.3389/fbioe.2020.00825)
Supplement: Supplementary file 1 [file Presentation_1.pdf]

## Supplementary Material

# 1 Supplementary Data

## 1.1 Supplementary Figures

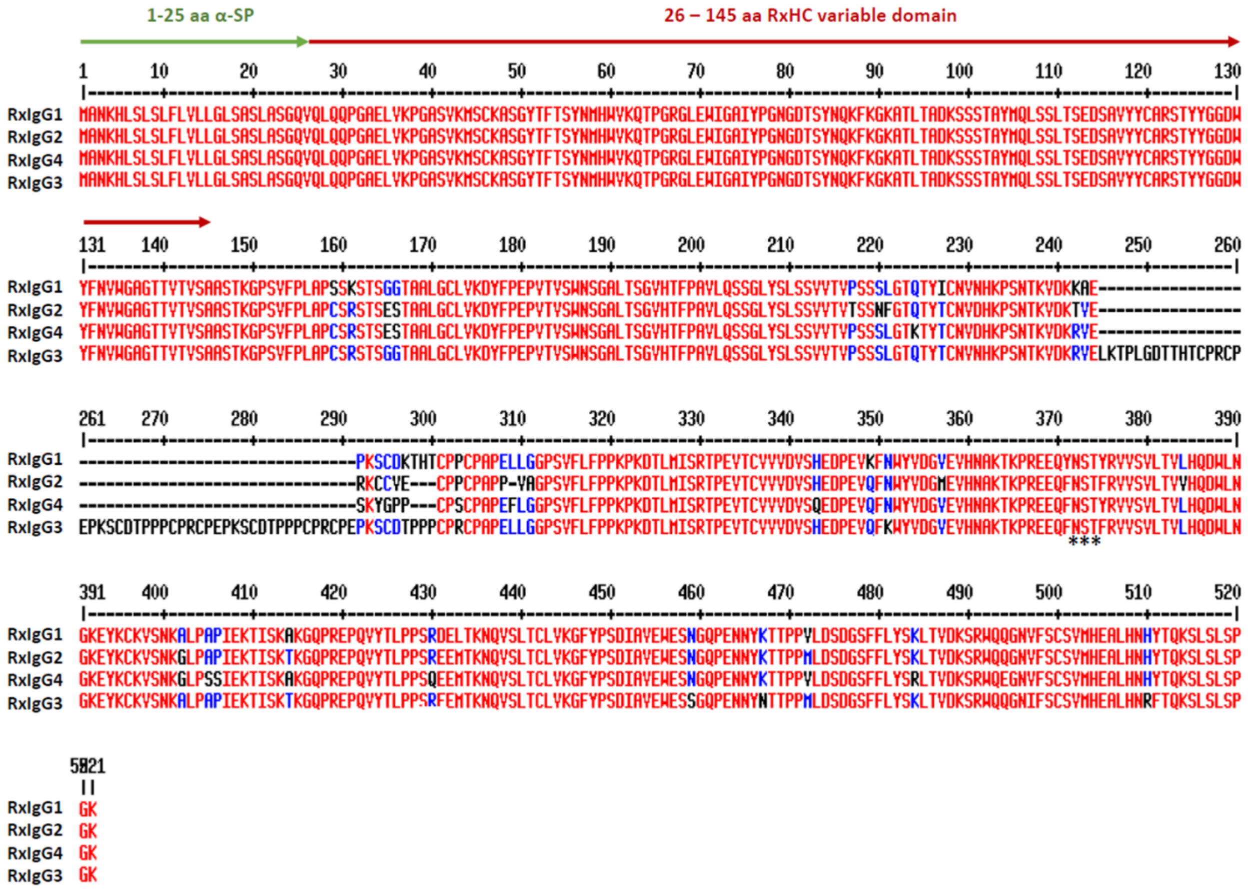

**Supplementary Figure 1 A:** Amino acid (aa) sequence of *RxIgGHC1-4*, including the α amylase signal peptide (α-SP, aa 1-25). AA 26-145 correspond to the variable heavy chain; Fc *N*-glycosylation site is indicated by asterisks.

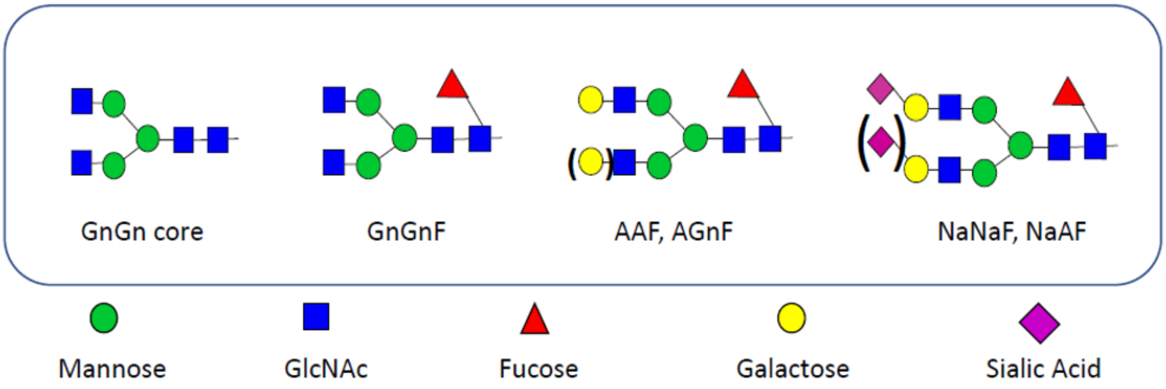


**Supplementary Figure 1 B:** Schematic presentation of N-linked Fc glycans frequently found on serum IgG: GnGn core structure is modified by core fucose (GnGnF) by one or two β,1,4 linked galactose (AAF structures also known as G1F, G2F) or by one or two sialic acid residues (NaNaF, also known as S1F, S2F) Nomenclature according to Consortium for Functional Glycomics (http://www.functionalglycomics.org)

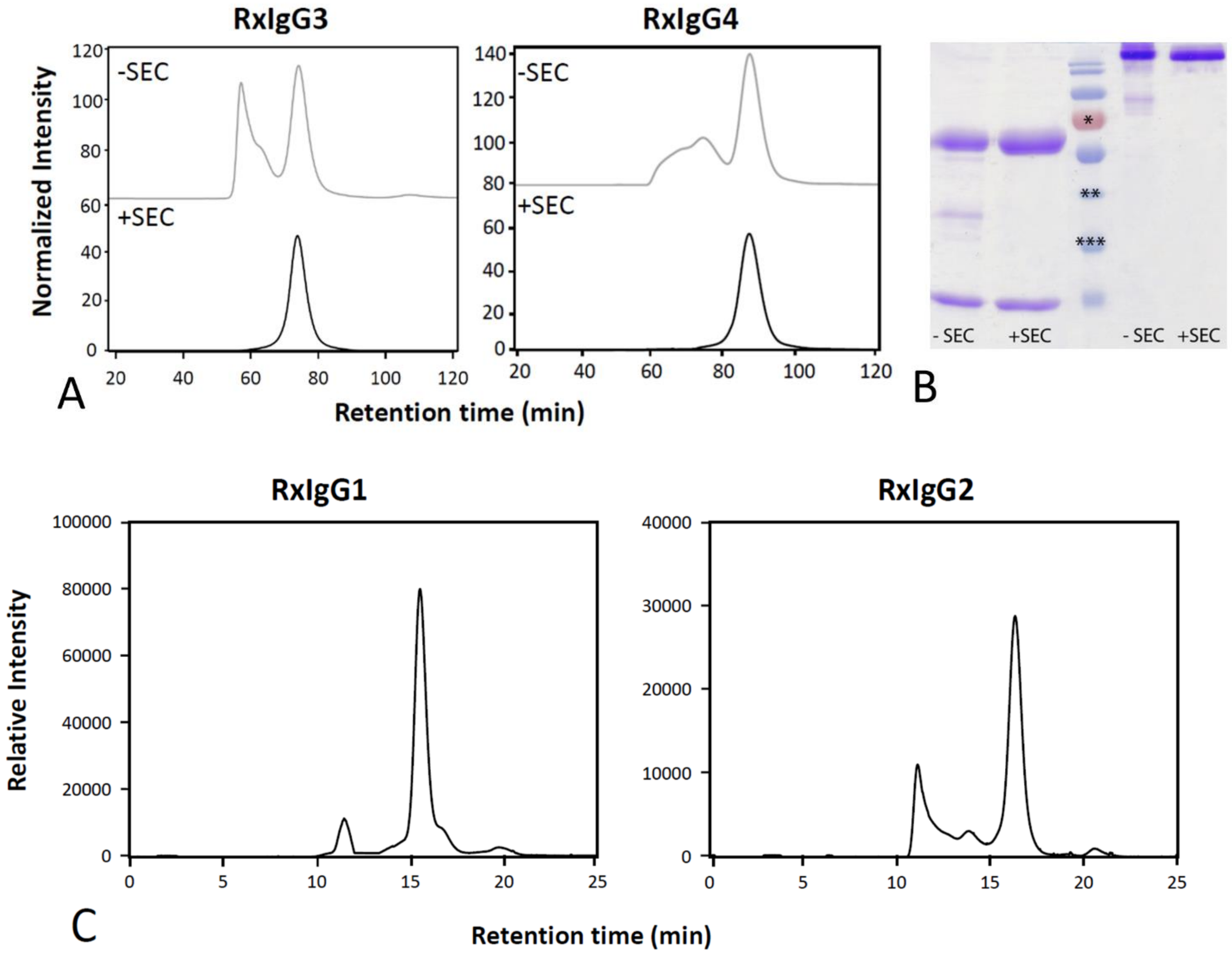


**Supplementary Figure 2: A:** Size exclusion chromatography profiles of RxIgG3 and RxIgG4 before (- SEC) and after (+ SEC). **B:** SDS-PAGE of IgG3 before (- SEC) and after (+ SEC); Left reducing, right non-reducing conditions. Marker: * 70, ** 45, *** 35 kDa (4µg IgG3 was loaded each line). C: SEC-HPLC-MALS profile of IgG1 and 2 (performed as described in Montero-Morales et al., 2019 doi:10.3389/fbioe.2019.00242)

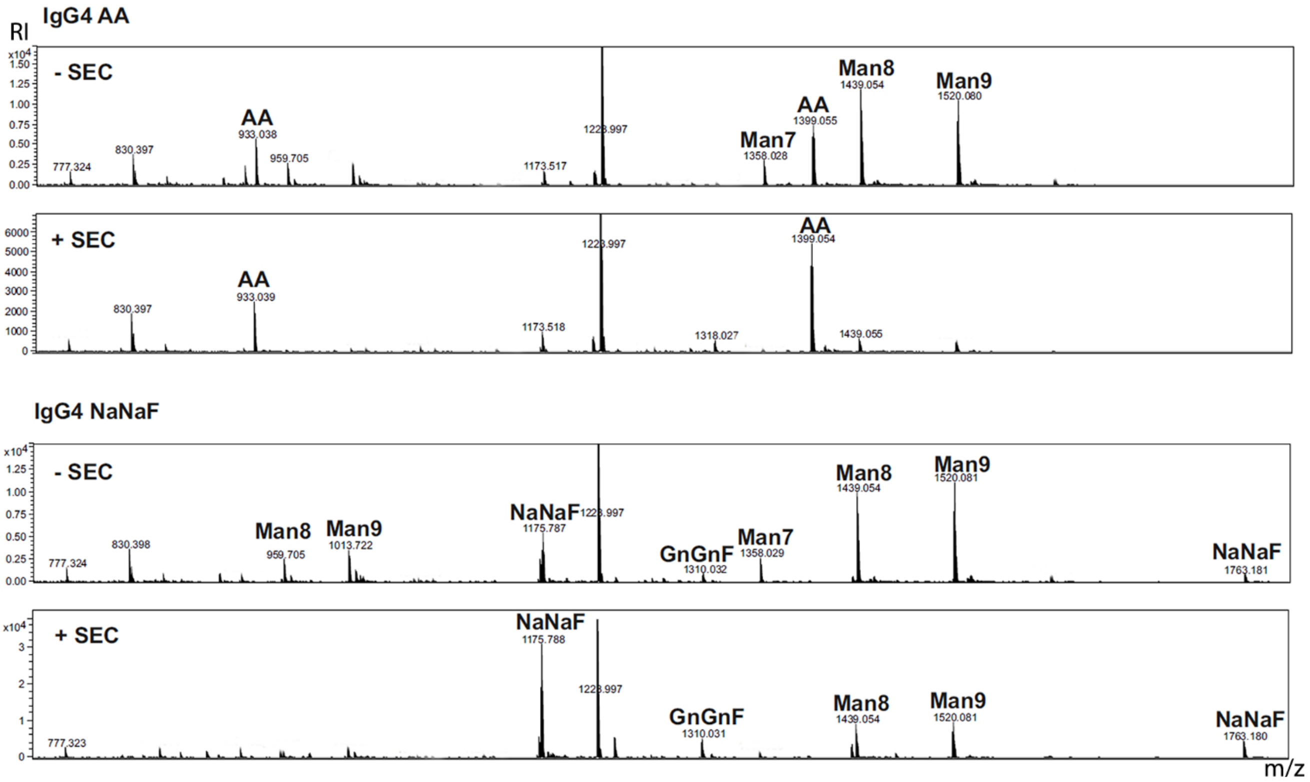


**Supplementary Figure 3:** LC-ESI-MS glycosylation profile of RxIgG4 AA and NaNaF before (-SEC) and after (+SEC) SEC. RI: relative intensity.
